# RNA sequencing-based analysis of the spleen transcriptome following infectious bronchitis virus infection of chickens selected for different mannose-binding lectin serum concentrations

**DOI:** 10.1186/s12864-016-2403-1

**Published:** 2016-01-27

**Authors:** Edin Hamzić, Rikke Brødsgaard Kjærup, Núria Mach, Guilietta Minozzi, Francesco Strozzi, Valentina Gualdi, John L. Williams, Jun Chen, Eva Wattrang, Bart Buitenhuis, Helle Risdahl Juul-Madsen, Tina Sørensen Dalgaard

**Affiliations:** UMR1313 Animal Genetics and Integrative Biology Unit, AgroParisTech, Université Paris-Saclay, 16 rue Claude Bernard, 75005 Paris, France; UMR1313 Animal Genetics and Integrative Biology Unit, INRA, Université Paris-Saclay, Domaine de Vilvert, 78350 Jouy-en-Josas, France; Department of Molecular Biology and Genetics, Center for Quantitative Genetics and Genomics, Aarhus University, Blichers Allé 20, P.O. Box 50, 8830 Tjele, Denmark; Department of Animal Science, Aarhus University, Blichers Allé 20, P.O. Box 50, 8830 Tjele, Denmark; Parco Tecnologico Padano, Via Einstein, 26900 Lodi, Italy; University of Milan, DIVET, Via Celoria 10, 20133 Milan, Italy; School of Animal and Veterinary Sciences, University of Adelaide, SA 5371 Roseworthy, Australia; Cobb-Vantress Inc, US-412 Road, Siloam Springs, AR 72761 USA; National Veterinary Institute, Ulls väg 2B, 751 89 Uppsala, Sweden

**Keywords:** IBV, Coronavirus, Infectious bronchitis, Chicken, RNA sequencing, Transcriptome, Spleen, Mannose-binding lectin, Immune response

## Abstract

**Background:**

Avian infectious bronchitis is a highly contagious disease of the upper-respiratory tract caused by infectious bronchitis virus (IBV). Understanding the molecular mechanisms involved in the interaction between innate and adaptive immune responses to IBV infection is a crucial element for further improvements in strategies to control IB. To this end, two chicken lines, selected for high (L10H line) and low (L10L line) serum concentration of mannose-binding lectin (MBL) were studied. In total, 32 birds from each line were used. Sixteen birds from each line were infected with IBV and sixteen were left uninfected. Eight uninfected and infected birds from each line were euthanized at 1 and 3 weeks post infection. RNA sequencing was performed on spleen samples from all 64 birds and differential gene expression analysis was performed for four comparisons: L10L line versus L10H line for uninfected birds at weeks 1 and 3, respectively, and in the same way for infected birds. Functional analysis was performed using Gene Ontology (GO) Immune System Process terms specific for *Gallus gallus*.

**Results:**

Comparing uninfected L10H and L10L birds, we identified 1698 and 1424 differentially expressed (DE) genes at weeks 1 and 3, respectively. For the IBV-infected birds, 1934 and 866 DE genes were identified between the two lines at weeks 1 and 3, respectively. The two most enriched GO terms emerging from the comparison of uninfected birds between the two lines were “Lymphocyte activation involved in immune response” and “Somatic recombination of immunoglobulin genes involved in immune response” at weeks 1 and 3, respectively. When comparing IBV-infected birds between the two lines, the most enriched GO terms were “Alpha-beta T cell activation” and “Positive regulation of leukocyte activation” at weeks 1 and 3, respectively.

**Conclusions:**

Healthy birds from the two lines showed significant differences in expression profiles for subsets of adaptive and innate immunity-related genes, whereas comparison of the IBV-infected birds from the two lines showed differences in expression of immunity-related genes involved in T cell activation and proliferation. The observed transcriptome differences between the two lines indicate that selection for MBL had influenced innate as well as adaptive immunity.

**Electronic supplementary material:**

The online version of this article (doi:10.1186/s12864-016-2403-1) contains supplementary material, which is available to authorized users.

## Background

Avian infectious bronchitis (IB) is an acute and highly contagious disease of the upper-respiratory tract caused by the infectious bronchitis virus (IBV). The virus is a member of the *Coronaviridae* family and has numerous serotypes and strains. Rapid replication combined with high mutation rate and recombination are the main causes of the observed high diversity [1]. The respiratory tract is the primary target organ and entry point for the virus, before further spread to kidneys and gonads. The most common symptoms of IB are related to the respiratory tract and include gasping, coughing, sneezing, tracheal rales, and nasal discharge [2]. Feed conversion and average daily gain are affected in broilers, and infection is often followed by secondary bacterial infections. In layers, IBV causes a reduction in egg production and egg quality. Today, IB is one of the most economically important diseases in the poultry industry [2].

Infection outbreaks are controlled by a combination of strict management practices and vaccination. The strict management practices, which include the maintenance of the housing temperature and ventilation, are essential, because IBV is highly contagious and spreads very fast. Live attenuated and inactivated vaccines are widely used for control and prevention of IBV infection [3, 4]. As there is little or no cross-protection between different serotypes/variants of the virus, hence vaccines should contain serotypes present in a particular area in order to induce adequate protection [1]. New multi-strain vaccines with the optimal antigen combination and optimal adjuvants are therefore required for future IBV control. Understanding the molecular mechanisms involved in the interaction between innate and adaptive immune responses to IBV infection is a crucial element for further improvements of the vaccines.

IBV infection induces a wide range of immune responses in chickens. An innate immune response is activated during the initial stages of infection in the mucosal lining of the trachea following binding of IBV virions to receptors on epithelial cells [5]. Activation of this innate immune response may be initiated by Toll-like receptor (TLR) signaling upon IBV recognition [6, 7]. In addition, rapid activation of natural killer (NK) cells has been observed one day after IBV infection [8] as well as increased macrophage numbers in lungs and trachea after primary IBV infection [9]. In the case of the adaptive immune responses, T lymphocyte subpopulations are actively involved in the early stages of IBV clearance [7, 10] exhibiting rapid activation upon IBV infection [6]. Furthermore, studies have shown that cytotoxic T lymphocytes (CTL) play an important role in responding to primary infections with IBV [10, 11]. In addition to T cell responses, IBV specific antibodies, of all three antibody classes present in chickens, have been reported [12–14]. A specific local antibody response in avian infectious bronchitis is characteristic for the response to a secondary infection [15]. The innate and adaptive immune systems are strongly interconnected, which is also seen in the response to IBV infection, and the connection possibly involves the serum collectin, mannose-binding lectin (MBL) as a key player [16].

Two chicken lines which were selected for high and low MBL serum concentrations (designated L10H and L10L, respectively), were used in the present study. Selective breeding has been performed for 14 generations using the combination of two strains (67.5 % UM-B19 chickens and 33.5 % White Cornish) as a starting population, as described by Juul-Madsen et al. [17]. The final result was two divergent lines, with mean MBL serum concentrations of 33.4 μg/ml for the L10H line and 7.6 μg/ml for the L10L line, respectively [18, 19]. The mean MBL serum concentration for 14 different chicken lines representing both broilers and layers is around 6 μg/ml, but varies from 0.4 to 37.8 μg/ml in normal healthy chickens with protein produced in the liver as the main source of circulating MBL [17]. In chickens, a positive correlation between MBL serum concentrations and the severity of several infections, such as infections caused by IBV [19], *Escherichia coli* [20] and *Pasteurella multocida* [21], has been observed. Chicken MBL binds to IBV [16, 22], therefore it is possible that MBL facilitates innate responses such as opsono-phagocytosis, complement activation or virus neutralization, in the early stages of IBV infection. In mammals MBL has also been shown to influence induction of adaptive immunity [23]. In support of the role of MBL in response to IBV, Kjaerup et al. [18] observed considerable differences in cellular adaptive immune parameters in response to an IBV infection between lines L10L and L10H. Furthermore, birds from L10H line exhibited lower viral loads and less severe damage of tracheal cilia following the IBV infection in comparison to birds from the L10L line.

The aim of this study was to characterize the spleen transcriptome of healthy birds from the two lines selected for serum MBL, and to investigate differences in molecular mechanisms behind the development of systemic adaptive immunity between the L10L and L10H lines infected with IBV.

## Results

### Animal experiment

**Fig. 1:**
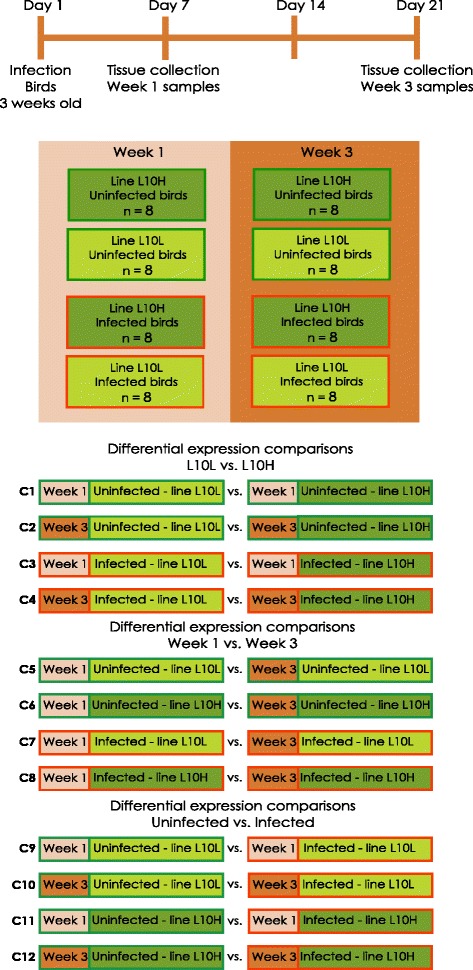
Structure and timeline of the experiment together with comparisons of gene differential expressions. The figure illustrates the experimental timeline together with the structure of the experiment. In total, 64 birds, 32 from each experimental line, L10H and L10L, were used. In addition, differential expression comparisons are shown

The experimental timeline and sampling time points are as illustrated in Fig. 1 and a full description of the experimental infection is reported by Kjaerup et al. [18]. The birds were infected at 3 weeks of age and from day 2 post-infection (p.i.), showed clinical signs characteristic of IBV infection, including sneezing and labored breathing. Viral loads in tracheal swabs were assessed for all birds as reported in the previous paper published on the experimental infection study [18] . No virus was detected in the uninfected birds at any time point throughout the experiment. Viral genomes were detected in swabs from infected birds from day 1 to 8 p.i. Notably, significantly lower viral loads (*p* < 0.03) were observed in birds from line L10H in comparison to infected birds from line L10L [18].

### Detection and quantification of splenic gene expression

RNA sequencing data were produced from eight infected and eight uninfected birds from each of the two lines at two sampling occasions, as described in the materials and methods section. All samples passed quality control measures for raw and trimmed sequenced reads except for individual no. 46, which was removed due to a very low number of sequenced reads. For the remaining birds, an average of over 37 million reads were obtained per sample for the 63 samples analyzed, with 81 % of the reads mapping to the chicken genome reference sequence, as described in the materials and methods section (See summary statistics with the number of mapped and total reads is presented in Additional file 1: Table S1). In total, 17,113 expressed genes were identified. After filtering genes with fewer than one read per million in eight samples [24] (genes which would not achieve statistical significance for differential expression), the final list contained 11,292 expressed genes. Before performing the differential gene expression analysis, further multivariate analysis was carried out on the raw and normalized gene count data to identify any discrepancies.

Multi-dimensional scaling (MDS) plot on expressed genes between the two lines, L10H and L10L, showed that they differ considerably in their transcriptome profiles for both uninfected and IBV-infected birds [See Additional file 2: Figure S1]. Moreover, inter-individual variation in gene expression at week 1 was considerably higher than that observed at week 3 for both uninfected and IBV-infected birds [See Additional file 2: Figure S1].

Birds 22 and 47 were separated from the rest on the MDS plot [See Additional file 2: Figure S1]. However, inspection of raw sequence data and mapping parameters did not identify any technical problems which would explain the observed out-grouping of these birds. In addition an interclass principal component analysis (PCA) was performed using raw and normalized gene counts. The interclass PCA revealed that the birds 22 and 47 were placed outside the 95 % confidence intervals of their respective treatments [See Additional file 3: Figure S2]. However, the PCA did not identify any gene having extreme count profiles which may have contributed to the transcriptome dispersion of birds 22 and 47 with respect to their treatment groups. Although there was no clear technical or biological explanation for their out-grouping, these samples were removed from further analysis.

### Identification of differentially expressed genes

Differential gene expression analysis was performed to compare the two chicken lines (L10L and L10H) at two time points for uninfected (C1 and C2, see Fig. 1) and IBV-infected birds (C3 and C4, see Fig. 1). A large number of genes were differentially expressed (DE) between L10L and L10H lines at weeks 1 and 3, for both uninfected and IBV-infected birds (see Table 1, see Fig. 1).

**Table 1:** Summary statistics of differentially expressed (DE) genes at FDR < 0.05

L10H versus L10L	Higher expression in L10H	No difference	Higher expression in L10L	DE-expressed
Uninfecteds at week 1 (C1)	692	9594	1006	1698
Uninfecteds at week 3 (C2)	774	9868	650	1424
Infected at week 1 (C3)	931	9358	1003	1934
Infected at week 3 (C4)	508	10426	358	866

Comparison between the two lines, L10H and L10L, uninfected and infected birds at two time points (weeks 1 and 3). Comparisons C1 – C4 correspond to differential gene expression comparisons presented in Fig. 1

We identified 1,698 and 1,424 DE genes for the uninfected birds between lines L10L and L10H at weeks 1 and 3, respectively (see Table 1). In total 692 genes had higher expression in L10H line and 1,006 had higher expression in line L10L for the uninfected birds at week 1 [See Additional file 4: Table S2] and 774 genes had higher expression in L10H line and 650 genes had higher expression in L10L line for uninfected birds at week 3 [See Additional file 5: Table S3].

Comparing IBV-infected L10H and L10L birds, we identified 1,934 and 866 DE genes at weeks 1 and 3, respectively (see Table 1). In total 931 genes had higher expression in line L10H and 1,003 had higher expression in line L10L at week 1 and at week 3, 508 had higher expression in line L10H and 358 had higher expression in line L10L (Table 1, Additional file 6: Table S4 and Additional file 7: Table S5).

**Fig. 2:**
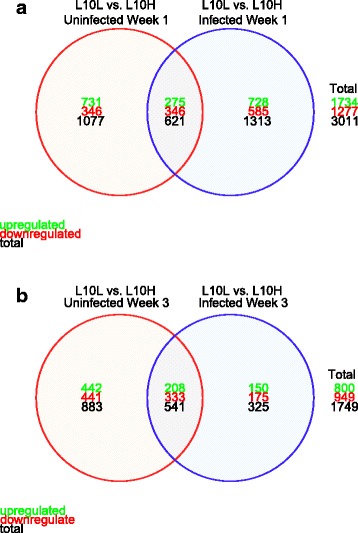
Venn diagram of differentially expressed (DE) genes between comparisons of uninfected and infected birds at different time points. **a** Differentially expressed (DE) genes between lines when the lines are uninfected (left circle) and infected (right circle). Numbers in the intersection correspond to DE genes that are in common between lines for uninfected and IBV infected birds at week 1. **b** DE genes between lines when the lines are uninfected (left circle) and infected (right circle). Numbers in the intersection correspond to DE genes that are in common between lines for uninfected and IBV infected birds at week 3. Numbers outside of circles represent sum of DE genes presented in circles

There were also status-related changes in gene expression as shown in the Venn diagram (Fig. 2). At week 1, the total number of DE genes in uninfected birds between the two lines was 1698 (1077 + 621) (Table 1, Fig. 2) which is lower comparing to 1934 (621 + 1313) DE genes in infected birds between the two lines (Table 1, Fig. 2). Out of 3,011 (1077 + 621 + 1313) DE genes for both uninfected and infected birds between the two lines only 621 (~20 %) were common for two comparisons (Fig. 2). At week 3, the total number of DE genes in uninfected birds between the two lines was 1424 (883 + 541) (Table 1, Fig. 2) which was higher comparing to 866 (541 + 325) in infected birds between the two lines (Table 1, Fig. 2). When comparing the uninfected and infected birds between the two lines, 541 (~30 %) genes were common out of total of 1749 (883 + 541+ 325) DE genes for both comparisons (Fig. 2).

Moreover, we also performed differential gene expression analysis to compare two time points (week 1 and week 3) in the two chicken lines (L10L and L10H) for uninfected (C5 and C6, see Fig. 1) and IBV-infected birds (C7 and C8, see Fig. 1). Finally, differential gene expression analysis was also conducted to compare the two infection states (uninfected and IBV-infected) at two time points for the L10L chicken line (C9 and C10, see Fig. 1) and the L10H chicken line (C11 and C12, see Fig. 1). As our main aim was to investigate differences between the two chicken lines, we only presented and discussed results related to the comparisons between the two chicken lines (C1 – C4, see Fig. 1). Results for the rest of comparisons (C5 – C12, see Fig. 1) were provided in the form of supplementary materials [See Additional file 8: Table S6, Additional file 9: Table S7, Additional file 10: Table S8, Additional file 11: Table S9, Additional file 12: Table S10, Additional file 13: Table S11, Additional file 14: Table S12 and Additional file 15: Table S13].

### Functional analysis of differentially expressed genes

**Fig. 3:**
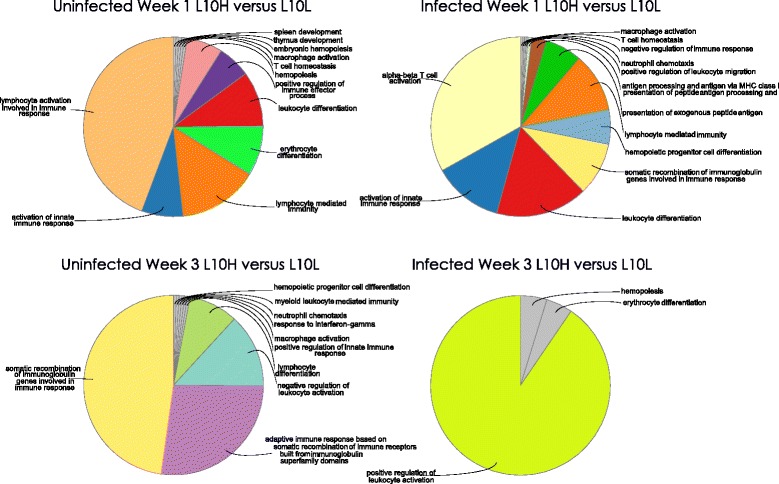
Functional map of differentially expressed genes enriched for GO Immune System terms. The top categories of the GO Immune System terms associated with differentially expressed (DE) genes. All categories were statistically significant (adjusted *p*-value < 0.001). The chart fragments represent the number of genes associated with the terms as a proportion of the total number of genes within the respective GO term. Terms which have not been grouped are shown in grey

An enrichment gene set analysis was carried out to identify over-represented Gene Ontology (GO) “Immune System Process” terms using the lists of DE genes from comparisons between uninfected and infected birds from the two lines at 1 and 3 weeks p.i. The most enriched GO Immune System terms between the two lines when comparing uninfected birds from the two lines and then infected from the two lines are shown in Fig. 3.

**Fig. 4:**
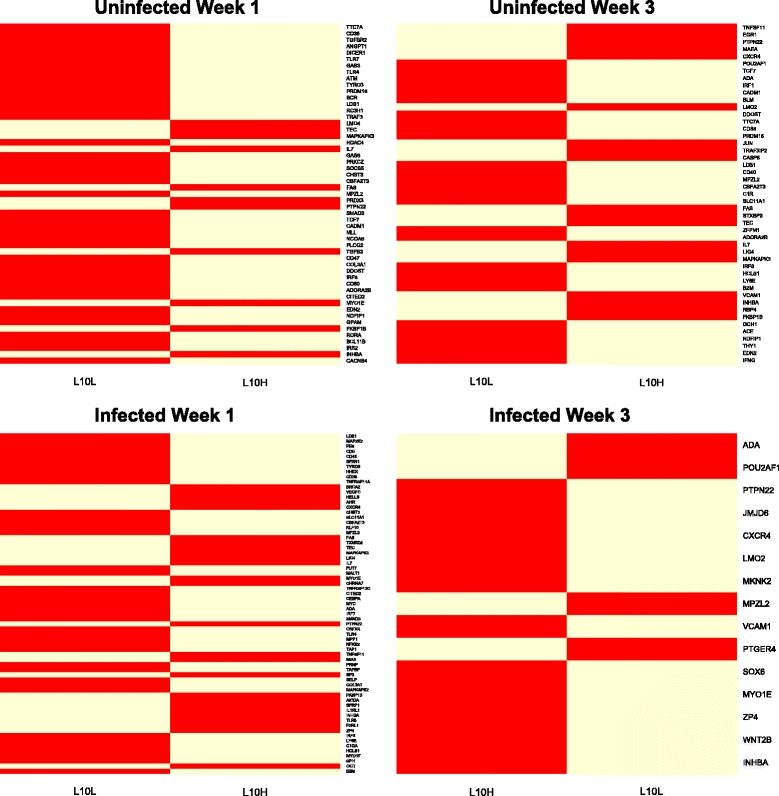
Differentially expressed genes associated with the GO Immune System term. Heatmap representation of the differentially expressed (DE) genes associated with the GO Immune System terms for the four comparisons between the two lines, L10H and L10L, uninfected and infected groups at two time points, weeks 1 and 3. The heat map is constructed using the average values of counts per millions for each group

GO Immune System terms associated with genes that were differentially expressed between the two lines for uninfected birds at week 1 were “Lymphocyte activation involved in immune response” (GO:0002285), “Activation of innate immune response” (GO:0002218), “Lymphocyte mediated immunity” (GO:0002449), and “Leukocyte differentiation” (GO:0002521) [See Fig. 3, Additional file 16: Figure S3]. In total, 53 DE genes were mapped to the GO Immune System terms (Fig. 4). Among the DE genes, *TGFB3* (Transforming growth factor beta 3), *IL7* (Interleukin 7), *FKBP1B* (FK506 binding protein 1B), *FAS* (Fas cell surface death receptor) and *PTPN22* (Protein tyrosine phosphatase, non-receptor type 22) had a higher expression in line L10H compared with L10L [See Fig. 4, Additional file 16: Figure S3]. Furthermore, the line L10H had a lower expression for a subset of innate immune genes: *TYRO3* (Tyrosine-protein kinase receptor 3), *TRAF3* (TNF receptor-associated factor 3) and *TLR7* (Toll-like receptor 7) compared with L10L [See Additional file 16: Figure S3, Fig. 4].

For uninfected birds at week 3, the most enriched GO Immune System terms were “Somatic recombination of immunoglobulin genes involved in immune response” (GO:0002204) and the “Adaptive immune response based on somatic recombination of immune receptors built from immunoglobulin superfamily” (GO:0002460) [See Fig. 3, See Additional file 17: Figure S4]. In total, 47 DE genes mapped to GO Immune System terms in this comparison (Fig. 4). Among the DE genes that had a higher expression in the line L10H at week 3, in the uninfected group, were *IL7*, *FKBP1B*, *FAS* and *PTPN22*, which were also seen differentially expressed between lines at week 1 [See Fig. 4, Additional file 17: Figure S4].

Comparing infected birds from the two lines, at week 1, “Alpha-beta T cell activation” (GO:0046631), “Activation of innate immune response” (GO:0002218) and “Leukocyte differentiation” (GO:0002521) functions were the three most enriched GO Immune System terms (Fig. 3). *CXCR4* (Chemokine receptor 4), *PTPN22* and *FAS* were among the most highly expressed genes in L10H [See Fig. 4, Additional file 18: Figure S5].

The major GO Immune System term that was strongly enriched for in the infected birds at week 3 was “Positive regulation of leukocyte activation” (GO:0002696) [See Fig. 3, Additional file 19: Figure S6]. Among the DE genes with higher expression in the L10H line were *CXCR4, VCAM1* (Vascular cell adhesion protein 1), *PTPN22* and *JMJD6* (Jumonji domain containing 6) [See Fig. 4, Additional file 19: Figure S6].

## Discussion

The present study used two lines, L10L and L10H, which have been divergently selected for high and low MBL serum concentration for 14 generations, respectively. These two lines have earlier been extensively used for immunological studies and exhibit differences in immunological parameters after being challenged with several pathogens [18, 22, 25].

The spleen is a secondary lymphoid organ where innate and adaptive immune responses can be efficiently mounted. In addition, the avian spleen is considered to play a very important immunological role because avian lymphatic vessels and lymph nodes are poorly developed [26]. The transcriptome differences in the spleen between the two lines, L10L and L10H, for uninfected (healthy) and IBV-infected birds were investigated, focusing on the differential expression of immune-related genes within significantly enriched immune-related GO terms. Large differences in transcriptome profiles were observed between birds from the two lines, both uninfected (healthy) and following the experimental IBV challenge [See Additional file 2: Figure S1]. This suggests that selection for MBL serum levels in the two lines had a much wider effect which goes beyond the expression of the MBL gene [27]. The observed transcriptome differences can probably be attributed to correlated response to selection [28] or random genetic drift [29]. Correlated selection occurs when a trait is affected by selection on a another trait and is dependent on a genetic correlation between the two traits, which is well known in animal breeding [30]. Alternatively, random genetic drift could contribute to the observed differences, considering that the founder population of the two lines was small [17].

Focusing on the expression of immune-related genes: at week 1, the uninfected birds showed differences in the expression of genes involved in both adaptive immunity and innate immunity-related pathways [See Additional file 16: Figure S3]. In addition, the line L10H had a lower expression for the subset of the innate immune genes, *TYRO3*, *TRAF3* and *TLR7* at week 1 [See Additional file 16: Figure S3 and Fig. 4]. *TYRO3* encodes tyrosine-protein kinase receptor 3 (TYRO3) which is involved in inhibition of TLR signaling pathways and TLR-induced cytokine signaling pathways. These two pathways influence immune-related processes, including cell proliferation/survival, cell adhesion and migration and inhibits the innate inflammatory response to pathogens [31]. *TRAF3* encodes a cytoplasmic signaling protein, which plays a critical role in the regulation of antiviral response and viral evasion [32–34]. *TLR7* was also among the DE innate immunity-related genes with higher expression in the line L10H. Chicken TLR7 has been shown to play a part in the response to IBV infections [9, 35].

In addition to the differences in the expression profiles for a subset of innate immune genes, the two lines also differed in their expression of adaptive immune genes. Uninfected birds from L10H had a higher gene expression compared to the line L10L, for *TGFB3*, *IL7*, *FKBP1B*, *FAS* and *PTPN22.* These genes are known to be involved in a wide range of adaptive immune processes. In humans, reducing the TGF-β signaling on T cells has been shown to increase the function of CD8 T cells in an indirect way which results in the rapid elimination of viruses, enabling the creation of an effective memory response [36]. Similarly, human IL-7 plays a key role in the survival of both naïve [37] and memory [38, 39] CD4 and CD8 T cells. Moreover, an *in vitro* study showed that inhibition of FKBP1B and other cyclophilins blocked the replication of different *Coronaviruses*, including IBV [40]. In analogy, uninfected birds from the L10H line had a higher expression of *IL7*, *FKBP1B*, *FAS* and *PTPN22*.

Generally the uninfected (healthy) birds from the two lines exhibit different expression profiles for this subset of innate and adaptive immune genes probably resulting from the divergent selection for the MBL serum concentration. Selection in animal breeding have been shown to have an extensive effect on a variety of traits including immunological [30]. Moreover, correlated response to selection has been observed in the case where selection was performed on less complex traits such as testosterone levels [41]. Finally, different expression profiles for the subset of innate and adaptive immune in the uninfected birds from the two lines might be due to the balance in the effect of MBL serum levels. High levels of human MBL have been mostly reported as beneficial while in case of intracellular parasitic disease the effect of MBL serum level might be opposite [42]. The results indicate that selection for MBL serum levels might lead to favoring specific modes of immune responses depending on the MBL function.

Large differences in the expression patterns were seen between the two lines following infection with IBV and these differences involved adaptive immunity-related pathways which are associated with “Alpha-beta T cell activation” (GO:0046631), and “Activation of innate immune response” (GO:0002218) [See Additional file 18: Figure S5]. The observed enrichment for GO terms related to T cell activation is in accordance with a previous study of these lines that showed that the IBV-specific T cells are present in large numbers in the spleen after IBV infection [43]. At week 1 post infection *CXCR4, FAS* and *PTPN22* showed higher expression in line L10H. The CXC chemokine receptors are expressed on both effector and memory T cells and play a key role in the homeostasis of memory T cells [44]. *FAS* has been shown to be upregulated in the kidney of chickens challenged with IBV [45]. The Fas/FasL pathway is an important pathway of killing for cytotoxic T cells [46]. Similarly, *PTPN22* has been shown to be differentially expressed in chickens following pathogen challenge, and in particular following infection with *Escherichia coli* [47]*.*

Additionally VCAM1 and *JMJD6* were among the adaptive immunity-related genes DE between lines at week 3 [See Fig. 4, Additional file 19: Figure S6]. The VCAM1 gene is known to be involved in the activation of T cells [48]. Furthermore, a recent study demonstrated that *JMJD6* regulates proliferation of memory T cells during a viral infection [49] which is of great interest considering that had higher expression in the IBV-infected birds from L10H line at week 3.

The results show that the two lines differ greatly in the expression of adaptive immunity-related genes following infection, which may imply the presence of different modes of gene regulation. The sampling times were chosen to access responses both in the effector phase (week 1) and memory phase (week 3) of the adaptive immune response to IBV. In accordance, at 1 week, post infection subsets of genes actively involved in T cell proliferation show differences between the lines. Also, at week 3 immune-related gene expression profiles in response to IBV infection that differ between the lines are more related to maintenance of T cell memory. MBL is known to be involved in regulation of dendritic cell maturation as well as cytokine production [50]. Dendritic cells, which are the main antigen presenting cells and are actively involved in regulation of adaptive immune responses, possess the receptors for MBL in mammals [23]. Therefore, the two lines selected for different MBL serum concentration may display differences in adaptive immune responses and development of adaptive immunity as a result of differences in response to cytokine signaling from dendritic cells. Other studies of the two lines, L10L and L10H, have shown that they differ in disease response parameters after being challenged with different pathogens. The L10L line has been associated with increased viral replication in the airway after an infectious bronchitis virus (IBV) infection [18], reduced growth rate after an *Escherichia coli* infection [20] and greater intestinal colonization after *Salmonella Infantis* infection [51]. In the present experiment, significantly lower viral loads (*p* < 0.03) were observed in birds from line L10H in comparison to infected birds from line L10L [18]. Furthermore, L10H birds in the present study exhibited a less severe damage of tracheal cilia following the IBV infection in comparison to the L10L line (unpublished data). In the current experiment phenotypic differences in additional traits connected to adaptive immunity were observed, including numbers of circulating B cells and cytotoxic T cells [18]. Based on these observations it seems that selection for high MBL serum concentration allows birds to cope better after being infected with a range of pathogens. Therefore, the observed differences in the expression profiles for the adaptive and innate immune-related genes are a reflection of differences in disease resistance and immune responses between the lines L10L and L10H.

## Conclusions

In conclusion, large differences in the spleen transcriptome between the two chicken lines, L10L and L10H, were observed in both uninfected (healthy) and IBV-infected birds. The uninfected birds from the two lines showed differences in expression profiles for a subset of both adaptive and innate immunity-related genes, which may represent differences in preparedness to respond to an infection. Following infection with IBV, the two lines showed large differences in expression of genes involved in the adaptive cellular immune response such as T cell activation and proliferation pathways and hence their ability to respond to the infection, which is reflected in the difference in pathogen load seen between the two lines.

## Methods

### Experimental design and tissue collection

This study is a follow-up of the experiment performed by Kjaerup et al. [18] which characterized the cellular and humoral immune response of the two chicken lines, L10H and L10L, divergently selected for MBL serum concentrations following IBV infection. In total, 96 birds were used in the experimental study originating from the two Aarhus University inbred lines, L10H and L10L [19]. All 96 birds were reared together in a biosecure IBV-free environment until they were 3 weeks of age and then allocated to two different groups with 24 birds from each line in each group (uninfected and infected). The birds were transferred to a biosafety level 2 facility and placed in isolators. Two isolators contained uninfected chickens and two isolators contained infected chickens. Each isolator having an equal mix of the two lines as described by Kjærup et al. [18].

The virulent IBV-M41 strain was used for the infection (a kind gift from Dr. med. vet. Hans C. Philipp at the Lohmann Animal Health GmbH, Cuxhaven, Germany). The virus had been passaged twice in specific pathogen-free embryonated eggs. The IBV inocula were prepared in phosphate-buffered saline (PBS) immediately before use and contained 2 × 10^5.2^ EID_50_ /200 μl of IBV-M41 virus. The first and the second group (the uninfected groups) were mock-infected with 200 μl PBS per bird. The third and fourth groups (the infected groups) received 200 μL of IBV-M41. The inocula were given half nasally and half orally to mimic the natural infection routes of IBV in the chicken. Chickens were fed diets that met or exceeded the National Research Council requirements. Feed and water were provided *ad libitum*. The birds were monitored daily for clinical signs of disease and disease parameters were measured as reported by Kjærup et al. [18]. None of the individuals received antibiotic therapy during the experimental period. The study was carried out under strict ethical approval and monitoring (see the statement at the end of the Materials and Methods section).

For this study 64 spleen samples were harvested and used for RNA sequencing. The birds were sacrificed 1 and 3 weeks post infection by cervical dislocation and spleen samples were collected. At both time points, eight samples from the two lines, L10H and line L10L, from each group (uninfected and infected) were collected as illustrated in Fig. 1. After collection, spleens were sectioned (triangular cross-sectional slice from upper part) and identical samples from each chicken were immediately placed in RNAlater® Stabilization Solution (Ambion Inc., Austin, Texas) that were incubated at 4 °C overnight and then transferred to – 20 °C the following day.

### RNA extraction and sequencing

Tissue samples were homogenized on a TissueLyzer LT (Qiagen, Hilden, Germany). Total RNA was extracted with the Qiagen RNAeasy Kit (Catalog ID 74104, Qiagen, Venlo, Netherlands) according to the manufacturer’s instructions. The quality of the 64 total RNA samples was verified using a 2200 TapeStation RNA Screen Tape device (Agilent, Santa Clara, CA, USA) and the concentration ascertained using an ND-1000 spectrophotometer (NanoDrop, Wilmington, DE).

Libraries were prepared with the Illumina TruseqRNA sample prep kit (Catalog ID FC-122-1001, Illumina, San Diego, USA) following the manufacturer’s protocol and evaluated with the Agilent Tape Station 2200. Libraries were quantified by Picogreen and then normalized to 10 nM as recommended by Illumina for cluster generation on the Hiseq2000. Equimolar amounts of each library were mixed before NaOH denaturation.

The Illumina Truseq PE cluster kit v3 (Catalog ID PE-401-3001) was used to generate clusters on the grafted Illumina Flowcell and the hybridized libraries were sequenced on six lines of a Flowcell on the Hiseq2000 with 100 cycles of a paired-end sequencing module using the Truseq SBS kit v3 (Catalog ID FC-401-3001).

### Quality control, mapping of RNA sequencing reads and counting mapped reads

Initial control quality was assessed by the FastQC software version 0.11.3 [52]. Raw reads were than trimmed for low quality bases using the Trimmomatic tool version 0.32 [53] applying minimum Phred quality score >10 averaged across the sliding window of five bases. Furthermore, all reads with the length below 40 bp were removed.

The trimmed reads were mapped to the *Gallus gallus* reference genome (Gallus_gallus-4.0, release 80 [54]) using a spliced aligner TopHat2 version 2.014 [55]. The *Gallus gallus* gene annotation used for mapping was retrieved from Ensembl database version 80 (www.ensembl.org). The mapping quality was assessed using a set of Python scripts within the RSeQC toolkit [56]. The quality control assessment included inspection of the read coverage over the full gene body in order to assess if reads coverage was uniform and if there was any 5’ or 3’ bias as well as how the mapped reads were distributed over genome features.

Gene count estimation was performed using the HTSeq-count tool in ‘union’ mode. The HTSeq-count is a Python script within the HTSeq framework, version 0.7.1, which is an open source toolkit that allows the input of raw counts from aligned reads to be annotated with gene names based on genomic features [57].

### Statistical analysis of the differential gene expression

The read counts obtained were used to estimate gene expression and identify differentially expressed (DE) genes. This was achieved using Bioconductor package edgeR version 3.10.0 [58] and limma version 3.24.5 [59] following a previously described protocol [24]. Before performing statistical analysis, genes with low levels of expression were filtered out using a threshold of least one read per million in *n* of the samples, where *n* is the size of the smallest group of replicates, which in this case was eight.

In order to account for technical and biological effects reads counts were normalized using the “calcNormFactors” function implemented in the edgeR package. This function normalizes the data by finding a set of scaling factors for the library sizes that minimizes the log-fold changes between the samples. The scale factors were computed using the trimmed mean of M-values (TMM) between samples [58]. Common and tag-wise dispersion estimates were calculated with the Cox-Reid profile adjusted likelihood method in order to correct for the technical and biological variation when fitting the multivariate negative binomial model [60].

Multidimensional scaling (MDS) was implemented in the edgeR package, to assess similarity of the samples visually. The MDS plot was created in order to visualize the relationship between samples and identify possible outliers [58]. MDS is based on comparing the relationship between all pairs of samples by applying a count-specific pairwise distance measure [58]. Possible outliers were further investigated using principal component analysis (PCA) to remove samples which fell outside a 95 % confidence ellipse.

A design matrix was created in order to specify the factors that were expected to affect the expression level. The matrix was constructed to fit the saturated model where each treatment combination was considered separately. Eight treatment combinations were considered as illustrated in Fig. 1: uninfected birds from the line L10H at week 1, uninfected birds from the line L10L at week 1, infected birds from the line L10H at week 1, infected birds from the line L10L at week 1, uninfected birds from the line L10H at week 3, uninfected birds from the line L10L at week 3, infected birds from the line L10H at week 3, infected birds from the line L10L at week 3.

A generalized linear model likelihood ratio test, specifying the difference of interest, was used to test for differential expression between these treatment combinations. The differential expression analysis was performed comparing the log-fold differences in gene counts between two lines (L10H and L10L) at different time points (weeks 1 and 3) and for different status (uninfected and infected) separately (Fig. 1). Benjamini Hochberg false discovery rates (FDR) for a transcriptome-wide experiment were calculated to correct for multiple testing [61]. All genes with an FDR-adjusted *p*-value <0.05 were considered individual genes of interest and were retained for further analysis.

## Functional analysis of differentially expressed genes

Functional analysis of the DE genes was performed using the Cytoscape version 3.2.1 [62, 63] with the ClueGo version 2.1.7 plug-in [64] to enrich the annotation and enrichment of the differentially expressed (DE) genes for four comparisons (C1-C4, see Fig. 1). ClueGO determines the distribution of the target genes across the GO (Gene Ontology) terms and pathways: this study focused on. The *p*-value was calculated using right-sided hypergeometric tests and Benjamini-Hochberg adjustment was used for multiple test correction. An adjusted *p*-value of 0.001 indicated a statistically significant deviation from the expected distribution, and that the corresponding GO terms and pathways were enriched for the target genes. The association strength between the terms was calculated using a corrected kappa statistic of 0.4. The network created represented the terms as nodes which were linked based on a 0.4 kappa score level. The size of the nodes reflected the enrichment significance of the terms. The network was automatically laid out using the Organic layout algorithm supported by Cytoscape. The functional groups were created by iterative merging of initially defined groups based on the predefined kappa score threshold. Only functional groups represented by their most significant term were visualized in the network providing an insightful view of their interrelations [64].

### Ethic statements

The experimental procedures were conducted under the protocols approved by the Danish Animal Experiments Inspectorate and complied with the Danish Ministry of Justice Law no. 382 (June 10, 1987) and Acts 739 (December 6, 1988) and 333 (May 19, 1990) concerning animal experimentation and care of experimental animals. The license to conduct the animal experiment was obtained by Helle R. Juul-Madsen.

### Consent for publication

Not applicable.

### Availability of supporting data

Raw and analyzed RNA-Seq data for this project have been deposited in GEO under accession code GSE73423 (http://www.ncbi.nlm.nih.gov/geo/query/acc.cgi?acc=GSE73423). Other supporting data are included as Additional files 1, 2, 3, 4, 5, 6, 7, 8, 9 and 10.

## Additional files

Additional file 1: Table S1. Summary of mapping statistics. Mapping statistics were obtained from TopHat2 and includes the number of mapped and total reads after FastQC and Trimmomatic quality control steps. Birds were assigned to eight different groups according to three levels: status (infected and uninfected), chicken line (L10L and L10H line) and time point (weeks 1 and 3). (XLSX 16 kb) Additional file 2: Figure S1. Multidimensional scaling (MDS) plot created using expression profiles of all normalized genes. Four MDS plots were created using the normalized gene count data set. The purpose of MDS plot is to provide a visual representation of the pattern of proximities (similarities or distances) among the set of studied birds. Plots are labeled by eight comparison groups and three different factor levels; status (uninfected and infected), line (L10L and L10H) and time (weeks 1 and 3). The plot was created using the “plotMDS.dge” function implemented in the edgeR package. In all plots, L10H_CTL_W1 (red, *n* = 8), L10H_CTL_W3 (blue, *n* = 7), L10H_INF_W1 (green, *n* = 8), L10H_INF_W3 (purple, *n* = 7), L10L_CTL_W1 (orange, *n* = 8), L10L_CTL_W3 (yellow, *n* = 8), L10L_INF_W1 (brown, *n* = 6), L10L_INF_W3 (pink, *n* = 8). (PDF 6 kb) Additional file 3: Figure S2. Interclass PCA of normalized gene count data. Interclass principal component analysis (PCA) with the eight comparison groups as the instrumental variable. The aim of interclass PCA was to identify any gene having extreme count profiles which may have contributed to the transcriptome dispersion of birds 22 and 47 with respect to their treatment groups. The plot was created using principal component analysis function implemented in the ade4 R package with birds as variables and differential expression comparison groups as class levels. (PDF 13 kb) Additional file 4: Table S2. Differentially expressed genes (FDR < 0.05) for comparison between L10L and L10H uninfected birds at week 1. (TXT 120 kb) Additional file 5: Table S3. Differentially expressed genes (FDR < 0.05) for comparison between L10L and L10H uninfected birds at week 3. (TXT 100 kb) Additional file 6: Table S4. Differentially expressed genes (FDR < 0.05) for comparison between L10L and L10H infected birds at week 1. (TXT 136 kb) Additional file 7: Table S5. Differentially expressed genes (FDR < 0.05) for comparison between L10L and L10H infected birds at week 3. (TXT 61 kb) Additional file 8: Table S6. Differentially expressed genes (FDR < 0.05) for comparison between Week 1 and Week 3 uninfected L10L birds. (TXT 444 kb) Additional file 9: Table S7. Differentially expressed genes (FDR < 0.05) for comparison between Week 1 and Week 3 uninfected L10H birds. (TXT 235 kb) Additional file 10: Table S8. Differentially expressed genes (FDR < 0.05) for comparison between Week 1 and Week 3 infected L10L birds. (TXT 161 kb) Additional file 11: Table S9. Differentially expressed genes (FDR < 0.05) for comparison between Week 1 and Week 3 infected L10H birds. (TXT 201 kb) Additional file 12: Table S10. Differentially expressed genes (FDR < 0.05) for comparison between uninfected and infected L10L birds at week 1. (TXT 213 kb) Additional file 13: Table S11. Differentially expressed genes (FDR < 0.05) for comparison between uninfected and infected L10L birds at week 3. (TXT 107 kb) Additional file 14: Table S12. Differentially expressed genes (FDR < 0.05) for comparison between uninfected and infected L10H birds at week 1. (TXT 88 kb) Additional file 15: Table S13. Differentially expressed genes (FDR < 0.05) for comparison between uninfected and infected L10H birds at week 3. (TXT 88 kb) Additional file 16: Figure S3. Network representation of enriched GO Immune System terms of differentially expressed (DE) genes for comparison between uninfected birds at week 1. The GO Immune System terms were identified as nodes and linked based on their kappa score level (> = 0.4) and *p*-value < 0.001. Functionally related groups partially overlapped. The GO terms are labelled in colors according to hierarchical clustering of GO terms. Terms which have not been grouped are shown in grey. The colour pie charts of the GO Immune system nodes show the gene proportion associated with the respective term. (PDF 60 kb) Additional file 17: Figure S4. Network representation of enriched GO Immune System terms of differentially expressed (DE) genes for comparison between uninfected birds at week 3. The GO Immune System terms were identified as nodes and linked based on their kappa score level (> = 0.4) and *p*-value < 0.001. Functionally related groups partially overlapped. The GO terms are labelled in colours according to hierarchical clustering of GO terms. Terms which have not been grouped are shown in grey. The color pie charts of the GO Immune system nodes show the gene proportion associated with the respective term. (PDF 55 kb) Additional file 18: Figure S5. Network representation of enriched GO Immune System terms of differentially expressed (DE) genes for comparison between infected birds at week 1. The GO Immune System terms were identified as nodes and linked based on their kappa score level (> = 0.4) and *p*-value < 0.001. Functionally related groups partially overlapped. The GO terms are labelled in colors according to hierarchical clustering of GO terms. Terms which have not been grouped are shown in grey. The color pie charts of the GO Immune system nodes show the gene proportion associated with the respective term. (PDF 70 kb) Additional file 19: Figure S6. Network representation of enriched GO Immune System terms of differentially expressed (DE) genes for comparison between infected birds at week 3. The GO Immune System terms were identified as nodes and linked based on their kappa score level (> = 0.4) and *p*-value < 0.001. Functionally related groups partially overlapped. The GO terms are labelled in colors according to hierarchical clustering of GO terms. Terms which have not been grouped are shown in grey. The color pie charts of the GO Immune system nodes show the gene proportion associated with the respective term. (PDF 30 kb)

## Abbreviations

IB: infectious bronchitis
IBV: infectious bronchitis virus
MBL: mannose-binding lectin
L10L: chicken line selected for low MBL serum levels
L10H: chicken line selected for high MBL serum levels
DE: differentially expressed
GO: gene ontology
TLR: toll-like receptor
NK: natural killer
MHC: major histocompatibility complex
PCA: principal component analysis
FDR: false discovery rate
PRR: pattern recognition receptors
TGF: tumor growth factor
PBS: phosphate-buffered saline
MDS: multidimensional scaling
TMM: trimmed mean of M-values
