# Supplementary figures and images for: RNA sequencing-based analysis of the spleen transcriptome following infectious bronchitis virus infection of chickens selected for different mannose-binding lectin serum concentrations
Source: BMC Genomics. 2016 Jan 27;17:82. doi: 10.1186/s12864-016-2403-1 (PMC4729133; doi:10.1186/s12864-016-2403-1)

**Uninfected – Infected, Week1 – Week 3,  
L10H–L10L**

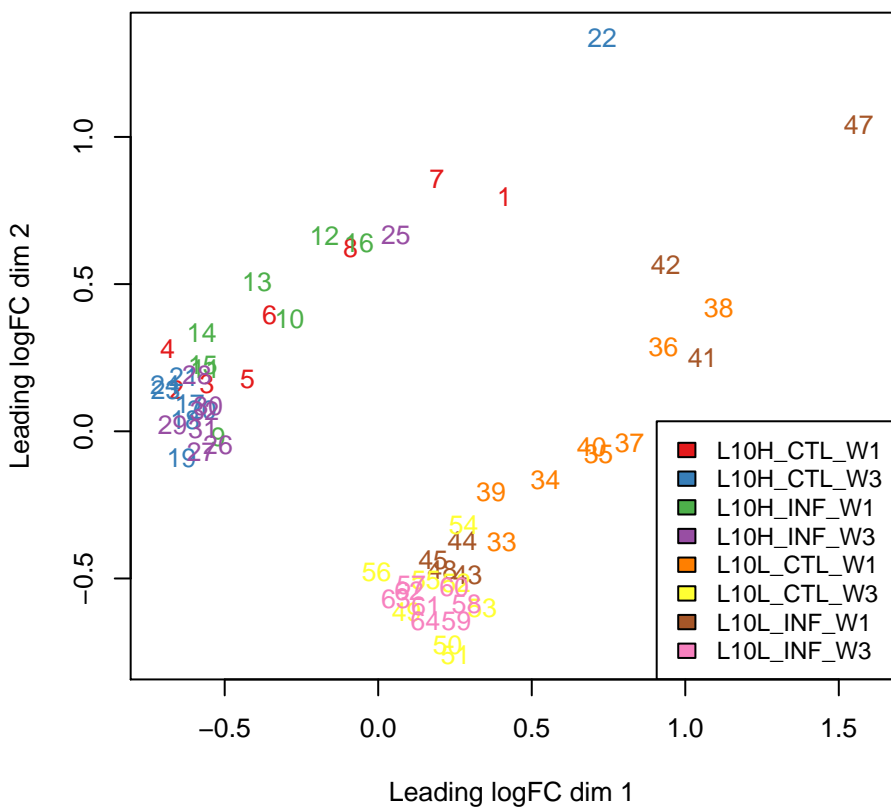

**Line L10H – Line L10L**

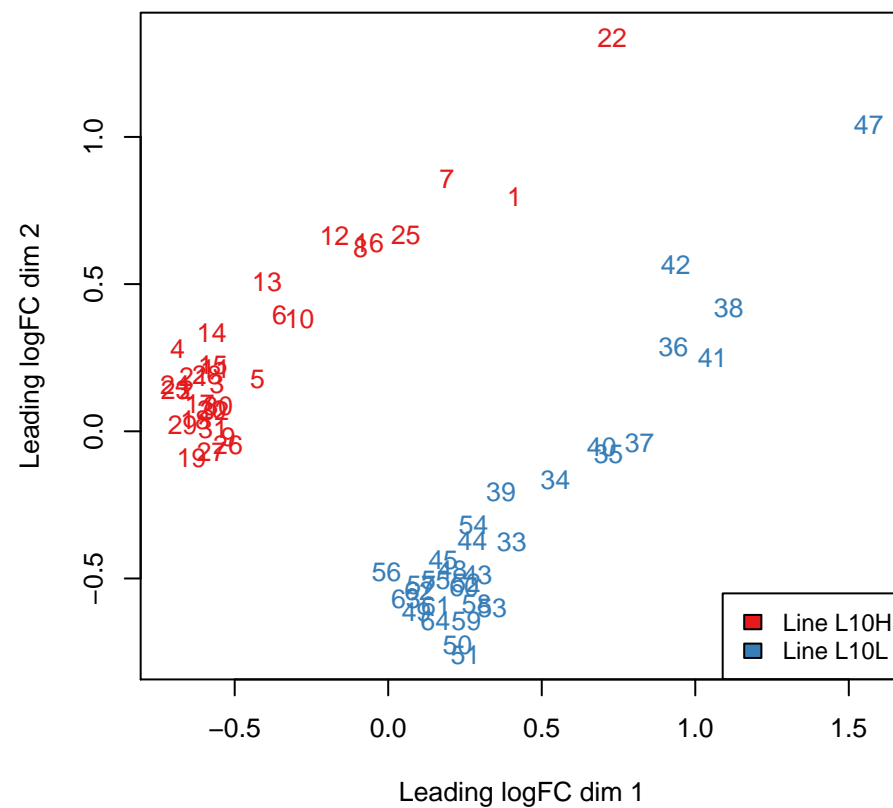

**Week 1 – Week 3**

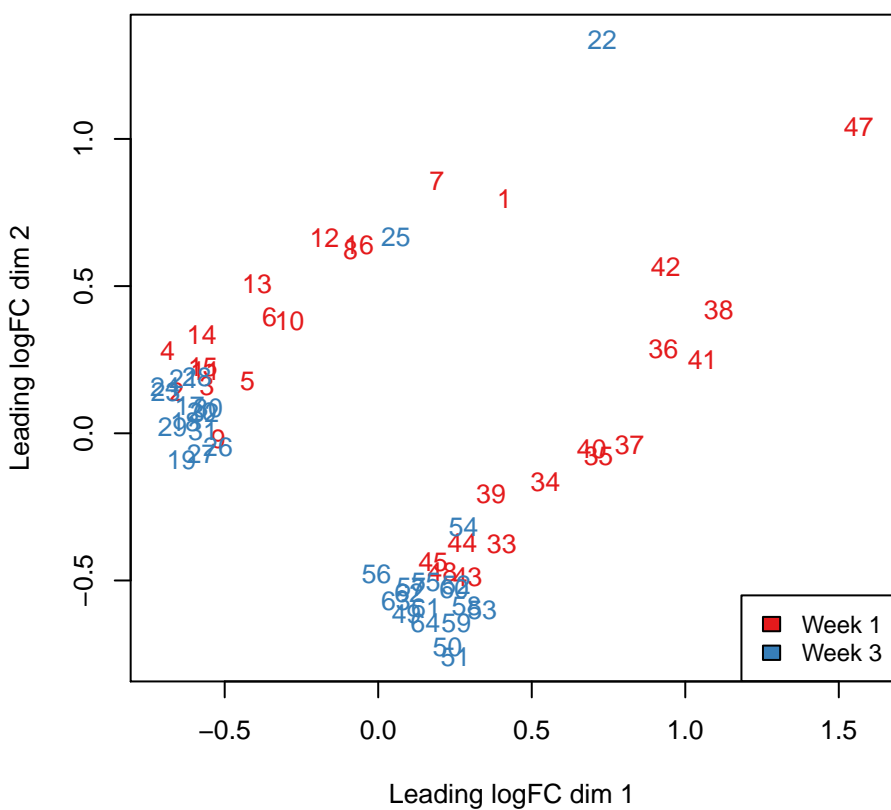

**Uninfected – Infected**

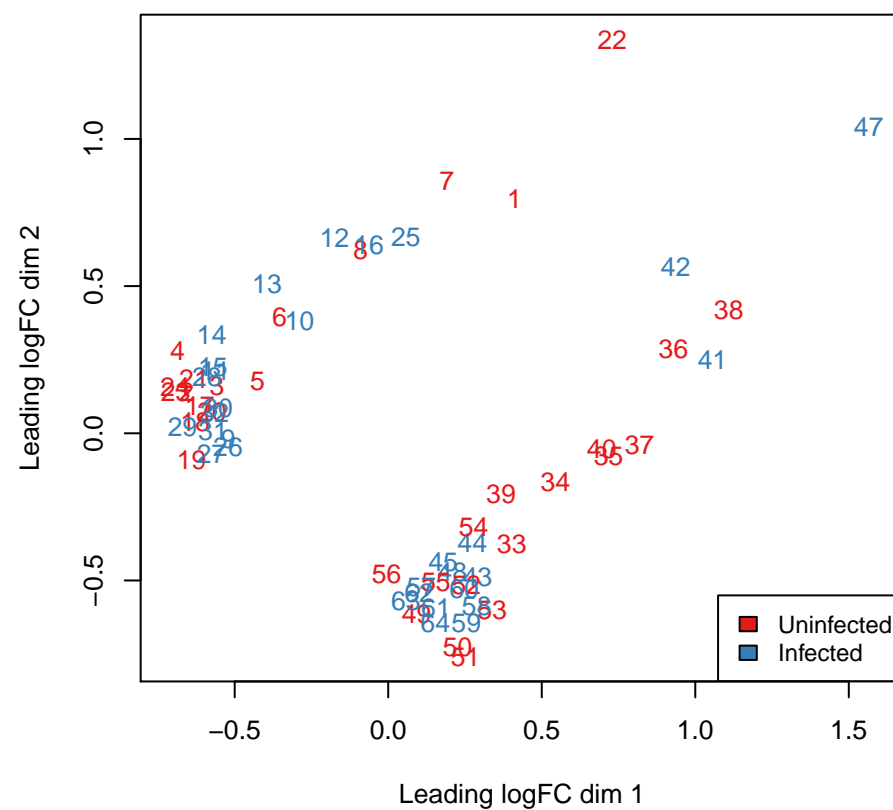

Supplement: Additional file 2: Figure S1. — Multidimensional scaling (MDS) plot created using expression profiles of all normalized genes. Four MDS plots were created using the normalized gene count data set. The purpose of MDS plot is to provide a visual representation of the pattern of proximities (similarities or distances) among the set of studied birds. Plots are labeled by eight comparison groups and three different factor levels; status (uninfected and infected), line (L10L and L10H) and time (weeks 1 and 3). The plot was created using the “plotMDS.dge” function implemented in the edgeR package. In all plots, L10H_CTL_W1 (red, n = 8), L10H_CTL_W3 (blue, n = 7), L10H_INF_W1 (green, n = 8), L10H_INF_W3 (purple, n = 7), L10L_CTL_W1 (orange, n = 8), L10L_CTL_W3 (yellow, n = 8), L10L_INF_W1 (brown, n = 6), L10L_INF_W3 (pink, n = 8). (PDF 6 kb) [file 12864_2016_2403_MOESM2_ESM.pdf]

d = 50

- L10H\_CTL\_W1
- L10H\_CTL\_W3
- L10H\_INF\_W1
- L10H\_INF\_W3
- L10L\_CTL\_W1
- L10L\_CTL\_W3
- L10L\_INF\_W1
- L10L\_INF\_W3

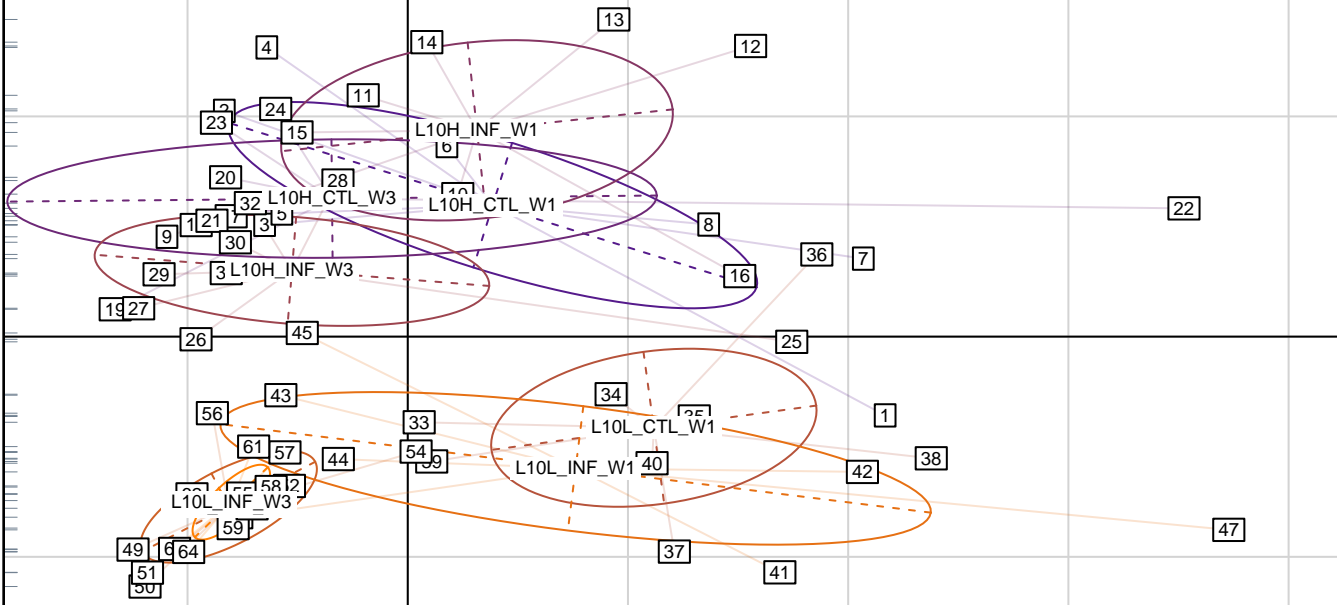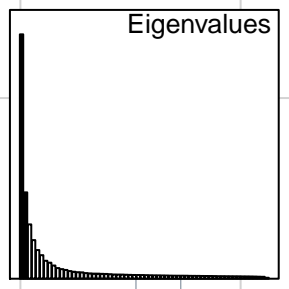

Supplement: Additional file 3: Figure S2. — Interclass PCA of normalized gene count data. Interclass principal component analysis (PCA) with the eight comparison groups as the instrumental variable. The aim of interclass PCA was to identify any gene having extreme count profiles which may have contributed to the transcriptome dispersion of birds 22 and 47 with respect to their treatment groups. The plot was created using principal component analysis function implemented in the ade4 R package with birds as variables and differential expression comparison groups as class levels. (PDF 13 kb) [file 12864_2016_2403_MOESM3_ESM.pdf]

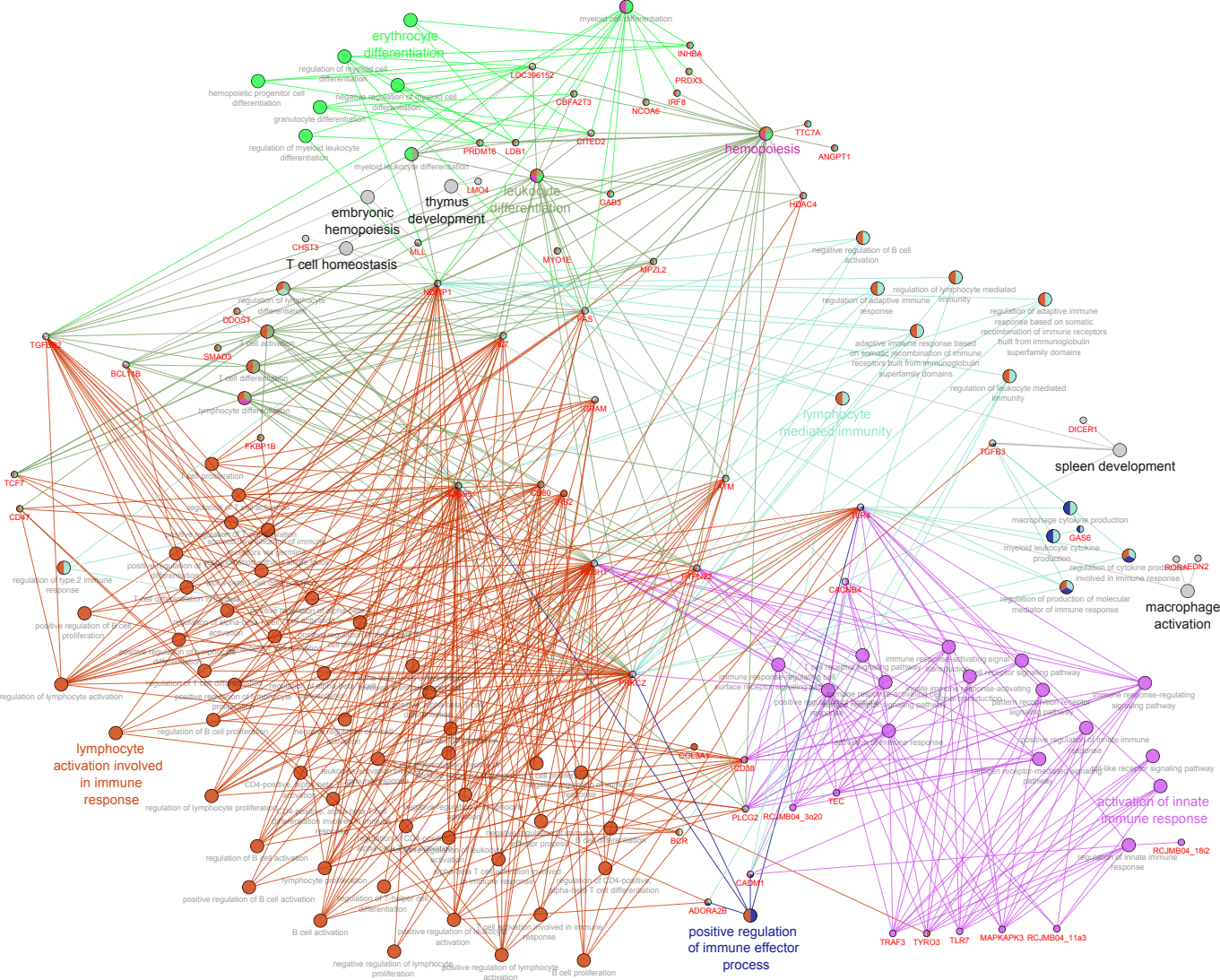

Supplement: Additional file 16: Figure S3. — Network representation of enriched GO Immune System terms of differentially expressed (DE) genes for comparison between uninfected birds at week 1. The GO Immune System terms were identified as nodes and linked based on their kappa score level (> = 0.4) and p-value < 0.001. Functionally related groups partially overlapped. The GO terms are labelled in colors according to hierarchical clustering of GO terms. Terms which have not been grouped are shown in grey. The colour pie charts of the GO Immune system nodes show the gene proportion associated with the respective term. (PDF 60 kb) [file 12864_2016_2403_MOESM16_ESM.pdf]

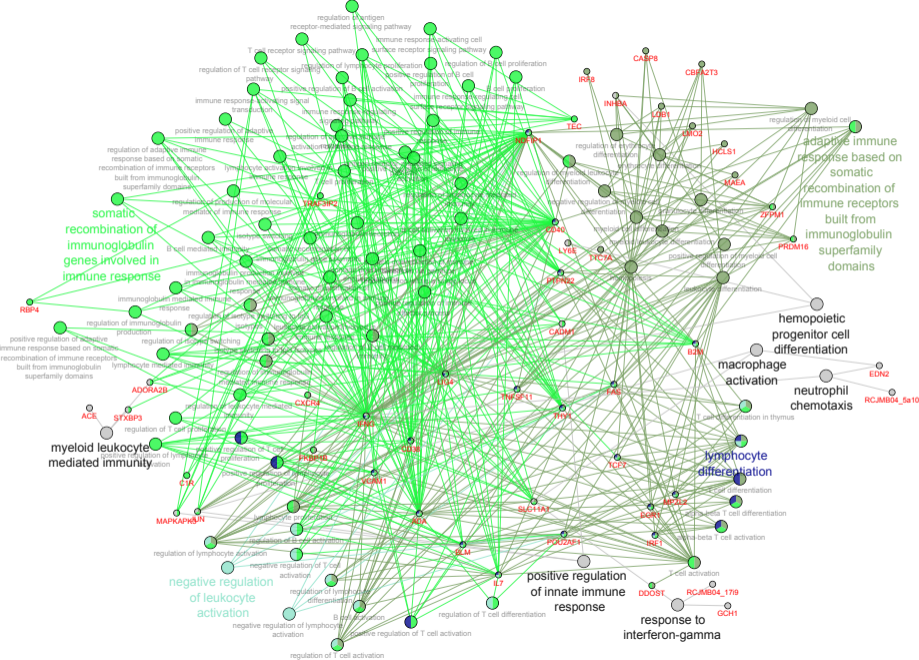

Supplement: Additional file 17: Figure S4. — Network representation of enriched GO Immune System terms of differentially expressed (DE) genes for comparison between uninfected birds at week 3. The GO Immune System terms were identified as nodes and linked based on their kappa score level (> = 0.4) and p-value < 0.001. Functionally related groups partially overlapped. The GO terms are labelled in colours according to hierarchical clustering of GO terms. Terms which have not been grouped are shown in grey. The color pie charts of the GO Immune system nodes show the gene proportion associated with the respective term. (PDF 55 kb) [file 12864_2016_2403_MOESM17_ESM.pdf]

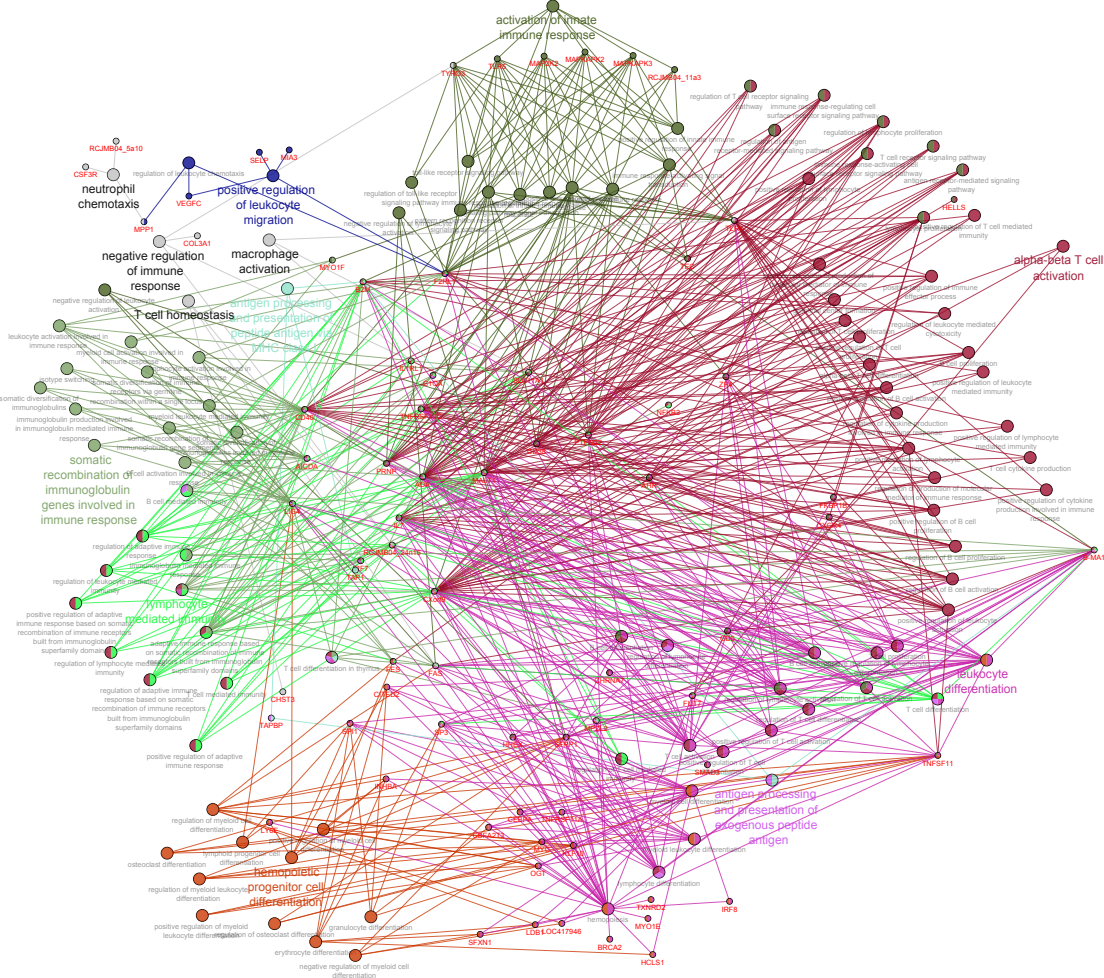

Supplement: Additional file 18: Figure S5. — Network representation of enriched GO Immune System terms of differentially expressed (DE) genes for comparison between infected birds at week 1. The GO Immune System terms were identified as nodes and linked based on their kappa score level (> = 0.4) and p-value < 0.001. Functionally related groups partially overlapped. The GO terms are labelled in colors according to hierarchical clustering of GO terms. Terms which have not been grouped are shown in grey. The color pie charts of the GO Immune system nodes show the gene proportion associated with the respective term. (PDF 70 kb) [file 12864_2016_2403_MOESM18_ESM.pdf]

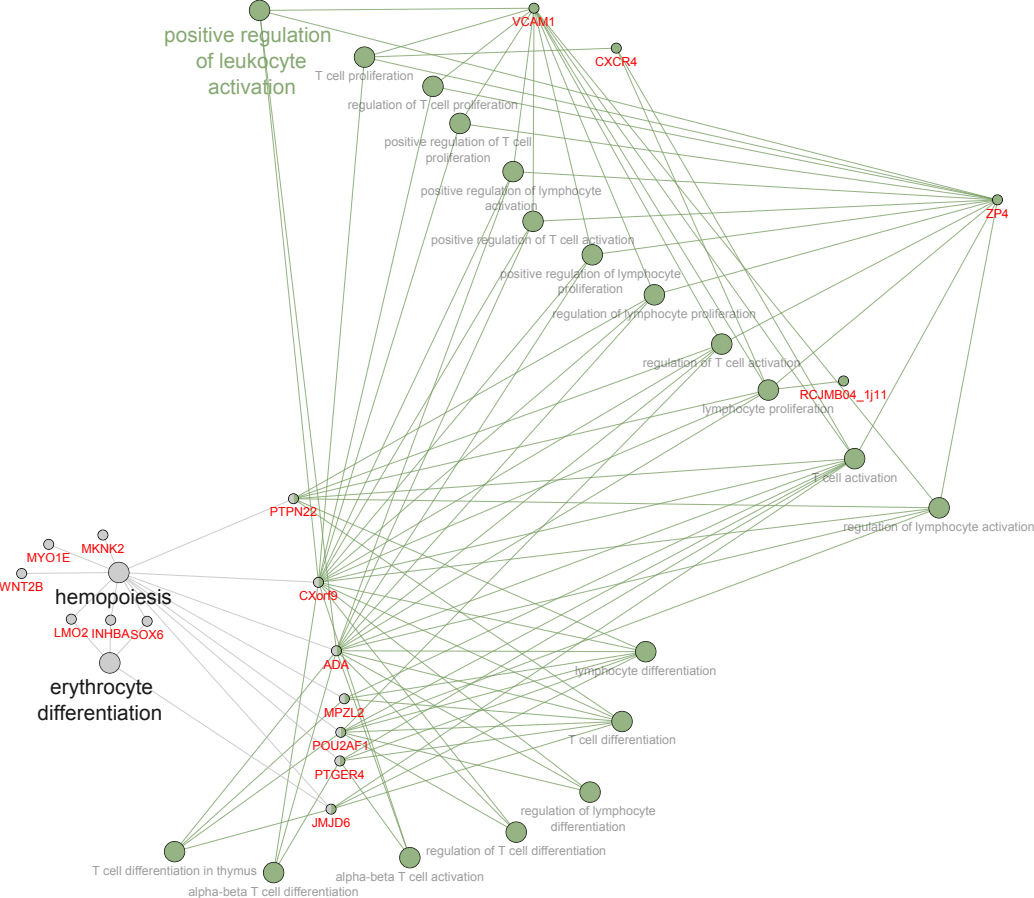

Supplement: Additional file 19: Figure S6. — Network representation of enriched GO Immune System terms of differentially expressed (DE) genes for comparison between infected birds at week 3. The GO Immune System terms were identified as nodes and linked based on their kappa score level (> = 0.4) and p-value < 0.001. Functionally related groups partially overlapped. The GO terms are labelled in colors according to hierarchical clustering of GO terms. Terms which have not been grouped are shown in grey. The color pie charts of the GO Immune system nodes show the gene proportion associated with the respective term. (PDF 30 kb) [file 12864_2016_2403_MOESM19_ESM.pdf]
